# Mesenchymal Stem Cell Transition to Tumor-Associated Fibroblasts Contributes to Fibrovascular Network Expansion and Tumor Progression

**DOI:** 10.1371/journal.pone.0004992

**Published:** 2009-04-07

**Authors:** Erika L. Spaeth, Jennifer L. Dembinski, A. Kate Sasser, Keri Watson, Ann Klopp, Brett Hall, Michael Andreeff, Frank Marini

**Affiliations:** 1 Section of Molecular Hematology and Therapy, Department of Stem Cell Transplantation and Cellular Therapy, The University of Texas M. D. Anderson Cancer Center, Houston, Texas, United States of America; 2 Department of Pediatrics, The Ohio State University and Center for Childhood Cancer, Columbus Children's Research Institute, Columbus, Ohio, United States of America; Ordway Research Institute, United States of America

## Abstract

**Background:**

Tumor associated fibroblasts (TAF), are essential for tumor progression providing both a functional and structural supportive environment. TAF, known as activated fibroblasts, have an established biological impact on tumorigenesis as matrix synthesizing or matrix degrading cells, contractile cells, and even blood vessel associated cells. The production of growth factors, cytokines, chemokines, matrix-degrading enzymes, and immunomodulatory mechanisms by these cells augment tumor progression by providing a suitable environment. There are several suggested origins of the TAF including tissue-resident, circulating, and epithelial-to-mesenchymal-transitioned cells.

**Methodology/Principal Findings:**

We provide evidence that TAF are derived from mesenchymal stem cells (MSC) that acquire a TAF phenotype following exposure to or systemic recruitment into adenocarcinoma xenograft models including breast, pancreatic, and ovarian. We define the MSC derived TAF in a xenograft ovarian carcinoma model by the immunohistochemical presence of 1) fibroblast specific protein and fibroblast activated protein; 2) markers phenotypically associated with aggressiveness, including tenascin-c, thrombospondin-1, and stromelysin-1; 3) production of pro-tumorigenic growth factors including hepatocyte growth factor, epidermal growth factor, and interleukin-6; and 4) factors indicative of vascularization, including alpha-smooth muscle actin, desmin, and vascular endothelial growth factor. We demonstrate that under long-term tumor conditioning *in vitro*, MSC express TAF–like proteins. Additionally, human MSC but not murine MSC stimulated tumor growth primarily through the paracrine production of secreted IL6.

**Conclusions/Significance:**

Our results suggest the dependence of *in vitro* Skov-3 tumor cell proliferation is due to the presence of tumor-stimulated MSC secreted IL6. The subsequent TAF phenotype arises from the MSC which ultimately promotes tumor growth through the contribution of microvascularization, stromal networks, and the production of tumor-stimulating paracrine factors.

## Introduction

Tumor cells are not self-sustaining entities, but interact via paracrine and juxtacrine signaling with the microenvironment. Among the cells found in this stromal environment are macrophages, endothelial cells, lymphocytes, fibroblasts, and pericytes which interact with tumor cells and the surrounding microenvironment through production of hormones, cytokines, chemokines and proteases. Tumors are thought to develop stroma from several sources, thus the characterization by Wels *et al.* of tumor-associated stromal cells as “migratory neighbors and distant invaders” [1]. Data in the literature currently support four origins: (1) the recruitment of resident tissue stem cells, (2) epithelial to mesenchymal transition of the tumor parenchyma, (3) fibroblast recruitment into the tumor stroma, and (4) recruitment of bone marrow-derived cells from the circulation [2]–[5].

We postulated that mesenchymal stem cells (MSC), like other bone marrow-resident cells, have the capacity to differentiate within the tumor microenvironment into fibroblastic-like cells that have been variably referred to as; myofibroblasts, tumor-associated (myo)fibroblasts (TAF), carcinoma-associated fibroblasts (CAF), fibrocytes or pericytes [6]. TAF have been shown to play an important role in tumor formation, growth and metastasis. The presence of fibroblast populations within human tumors is associated with poor outcome and an increase in metastatic potential [7], [8]. These TAF are associated with expression of factors involved in degradation of matrix proteins, angiogenesis and promotion of cell growth: matrix metalloproteinases (MMP), plasminogen activator inhibitor-1, vascular endothelial growth factor (VEGF), insulin growth factor (IGF-2) and hepatocyte growth factor (HGF) [9]. The TAF population differs from a normal fibroblastic phenotype because of its rich source of tumor-growth-promoting factors, pro-angiogenic factors and expression of myofibroblastic characteristics. TAF are characterized by the presence of four qualifying factors: (1) fibroblast markers fibroblast-specific protein (FSP) and fibroblast activating protein (FAP); (2) genes associated with an increase of tumor aggression, including stromelysin-1 (SL-1), thrombospondin-1 (Tsp-1) and tenascin-C (Tn-C); (3) myofibroblast/provascularizing potential including desmin, alpha-smooth muscle actin (α-SMA), and vascular endothelial growth factor (VEGF) [10]; and lastly, (4) growth factors, transforming growth factor-beta (TGF-β), HGF/scatter factor (SF), basic fibroblast growth factor (bFGF) and epidermal growth factor (EGF).

MSC comprise a unique population of cells utilized for tissue maintenance and repair; their regenerative ability and multipotent capacity allow for their differentiation into osteocytes, adipocytes, chondrocytes, or myocytes. Importantly, MSC possess an innate tropism for injured tissue. Our group originally demonstrated the capacity of MSC, also known as multipotent stromal cells, to home to tumors and participate in tumor stroma formation, suggesting MSC as a potential source of stroma [11], [12]. MSC are a heterogeneous population of connective tissues progenitors found in many locations, such as adipose tissue, dermis, and bone marrow. The tropism of MSC for tumors is thought to be due to similarity in factors secreted by wounds and tumors [13], substantiating the concept that tumors are “wounds that never heal” [14], [15]. It has been proposed that this tropism could be exploited by using MSC as gene-delivery vehicles for anticancer therapies, and this approach has shown promise in preclinical models [11], [12], [16]–[19]. The ability of MSC to travel to solid tumors after intravenous administration and their ability to develop myofibroblast-like characteristics under defined culture conditions [20] both suggest that TAF could be derived from MSC.

Our study was designed to assess whether MSC are precursors of TAF-like cells in tumors and to analyze the contribution of MSC to the growth and development of tumor fibrovascular networks. We show herein the propensity of MSC to transition within a xenograft tumor model to TAF-like cells and, by immunohistochemical (IHC) staining, their tumor-supportive nature. In a murine tumor xenograft model, human tumors with admixed human MSC demonstrated cells expressing activated fibroblast-like markers including FAP, FSP, α-SMA, Tn-C, Tsp-1, desmin or SL-1, while tumors without admixed MSC did not show these markers. The presence of MSC within the tumor also enhanced expression of growth factors such as EGF and HGF. We further demonstrate that the microenvironment provided by the tumor stimulated MSC directly to enhance the growth of Skov-3, which is significantly enhanced by growth factors interleukin-6 (IL-6) and EGF.

Our data suggest the involvement of the MSC as a component of tumor associated fibrovascular networks—this includes the pericytic population that contributes to the microvessel involved in the neovacularization as well as the fibroblastic population that contributes to matrix remodeling and tumor growth. To our knowledge is the first-in-kind report to demonstrate four levels of tumor support by MSC in the tumor microenvironment: 1) matrix formation, 2) growth factor production, 3) vasculogenesis/angiogenesis and 4) expression of proteins associated with tumor aggression.

## Results

### Defining TAFs in the context of MSC

**Figure 1:**
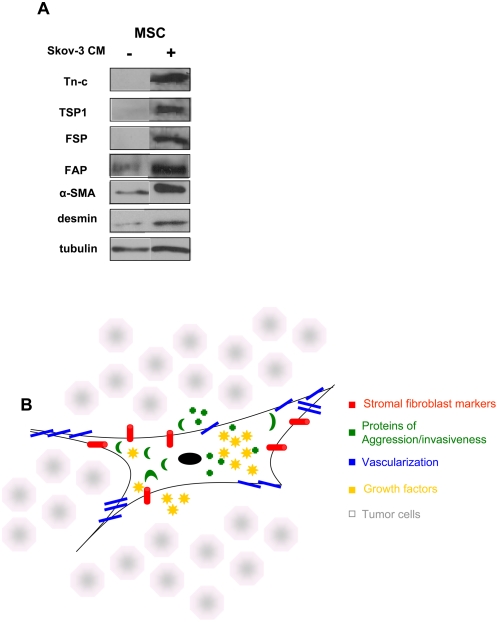
In admixtures of tumor and MSC, MSC exhibit four attributes of TAF. (A) Initially, MSC do not express all TAF markers. As shown by western, huMSC, prior to Skov-3 tumor exposure, express low levels of α-SMA, FAP and desmin and are negative for the expression of, FSP, TSP-1, and Tn-C. Following 16 days exposure to Skov-3-conditioned medium, the MSC express Tn-C, TSP-1, FSP and increase expression of α-SMA, FAP and desmsin. (B) The TAF is a fibroblastic cell that has four defining characteristics, which together, distinguish it from the normal fibroblast: The fibroblastic nature of the cell (*red;* Fibroblast activation protein and fibroblast specific protein); the aggressive/invasive nature of the cell defined by the secreted proteins (*green;* TSP-1, Tn-C and SL1); the vascularization potential of the cell (*blue;* α-SMA, desmin and VEGF); and the growth factors secreted by the cell to aid in tumor growth and development (*yellow;* EGF, HGF, IL-6 and bFGF).

The MSC population used in our investigation was characterized by flow cytometry and by western blot, prior to our *in vivo* analysis of the MSC. We were able to induce a TAF phenotype *in vitro* by conditioning MSC with Skov-3 conditioned medium over a period of 16 days. Naïve MSC expressed low levels of α-SMA, FAP and desmin and did not express the TAF markers FSP, TSP-1 or Tn-C (**Figure 1A**): they were positive for the MSC markers CD105, CD90, CD44, CD146, CD140b and CD166 and negative for hematopoietic and endothelial markers CD31, CD34 and CD45 (**Figure S1**). Following 16 days of *in vitro* conditioning with Skov-3 cell medium the MSC express high levels of Tn-C, TSP and FSP and increased their expression of α-SMA, FAP and desmin.

We define a TAF by four characteristics that are outlined in **Figure 1B**, these include fibroblast surface proteins (FAP and FSP), proteins indicative of invasion and remodeling (TSP1, Tn-C, SL-1), proteins associated with neovascularization (α-SMA, desmin and VEGF), and tumor promoting growth factors (HGF, EGF and IL6). In the following sections, we provide *in vivo* evidence of the expression of these factors only in tumors that have an admixed population of MSC, thereby illustrating the MSC involvement as a TAF within the tumor microenvironment.

### TAF marker expression in tumors harboring MSC

To elucidate the role that MSC play within the tumor microenvironment, we focused on the MSC once engrafted into the tumor. huMSC were admixed at a 1∶1 ratio with Skov-3 tumor cells prior to intravenous injection. Tumor growth was monitored for 91 days after injection of the admixed, or Skov-3 only cells. The tumors were removed from the mice, snap frozen and sent for tissue processing at day 91 (see methods section). To support our hypothesis that MSC contribute to the TAF population within the tumor microenvironment, we stained the admixed and Skov-3 only tumors sections for four different TAF characteristics: fibroblast surface markers, markers of vascularization, markers of invasiveness and aggressiveness and expression of tumor-promoting growth factors (**Figure 1B**).

**Figure 2:**
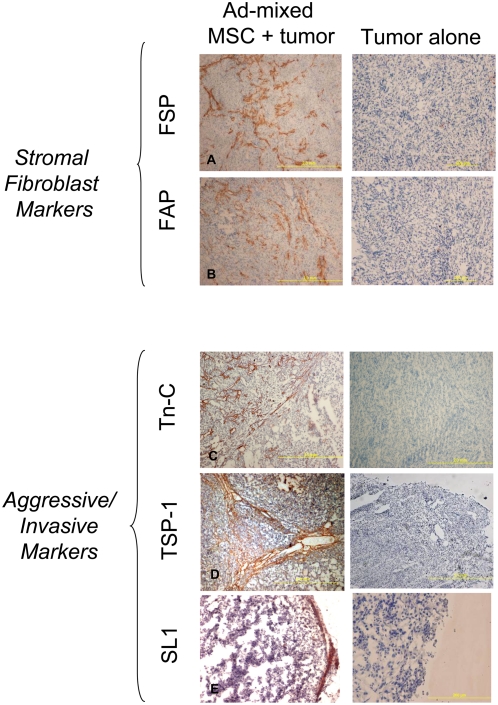
Admixed Skov-3/MSC xenografts express fibroblast markers and invasive markers indicative of TAF. IHC staining for markers that define fibroblast presence are visible in the Skov-3/MSC (1∶1) admixed xenografts but not in Skov-3-only xenografts. Fibroblast markers FAP (A) and FSP (B) are both present throughout the admixed tumors. The three tissue remodeling proteins Tn-C (C), TSP-1 (D) and SL-1 (E) are detectable within the admixed tumors. These two categories cover the first two characteristics of a TAF, and are observed only in tumors exposed to MSC. Human-specific, mouse non-cross-reactive antibodies allow identification of the human MSC-contributory components within the tumor microenvironment.

IHC of fibroblast surface protein expression was conducted on tumor sections to identify the first TAF characteristic. FSP and FAP staining was evident within the admixed tumors that received MSC but not in the untreated controls (**Figure 2A and 2B**). The FAP and FSP are not ubiquitously expressed in the stroma, but are restricted to cells that we identify as TAF and are stained in an unorganized, unparallel striated pattern. The admixed tumors have, by day 91, a larger area of human stromal contribution compared to the Skov-3 only tumors. While the murine stromal contribution is present in all the tumors, it is negligible compared to the human stromal component of the admixed Skov-3/MSC tumors (data not shown).

We then examined the expression of proteins consistent with an aggressive or invasive phenotype. Three molecules that are vital to the pathophysiological extracellular matrix (ECM) structure, Tn-C, Tsp-1 and SL-1, are secreted by fibroblast-like cells and modulate the structural architecture of the tumor. While MSC do not typically express these markers, the expression of all three proteins was evident once the MSC were within the tumor microenvironment. Both TSP-1 and Tn-C expression patterns were high throughout the tumor microenvironment (**Figure 2C and 2D**). The staining of the TSP-1 and Tn-C associate with many of the neovascular and microvascular lumen within the tumor, a phenomenon consistent with one of the TAF cell phenotypes, the pericyte. The presence of SL-1 was observed primarily on the leading edge of the tumor mass consistent its function as a proteolytic enzyme (**Figure 2E**). These markers were not expressed, however, in Skov-3 tumors that had not been admixed with MSC.

**Figure 3:**
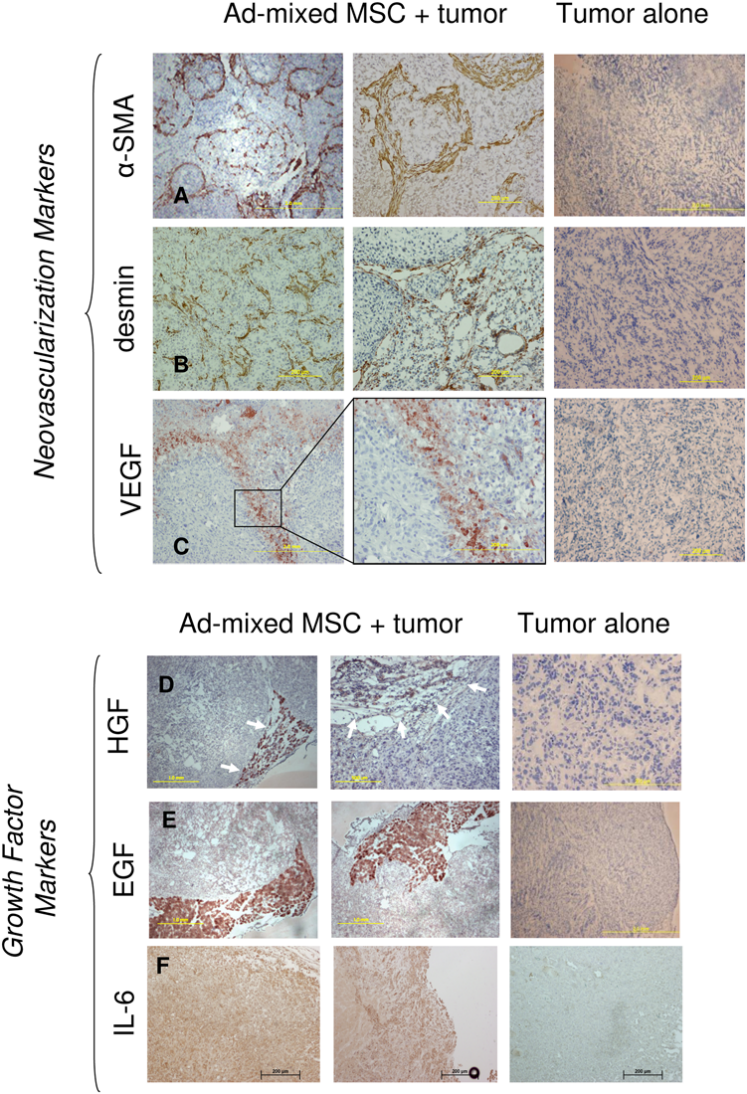
Admixed Skov-3/MSC xenografts express provascularization markers. One characteristic of the TAF is their ability to form fibrovascular networks, which include vessels and the architectural foundation in which they lay. IHC staining reveals structural proteins α-SMA (A) and desmin (B) as well as factors suggestive of tumor vascularization, including VEGF (C) in the presence of admixed MSC but not in tumors that received no MSC (far right panel). HGF (D) is found within the stromal compartment of the tumor. A strong delineation separating the tumor and stromal compartments is formed when the admixed xenograft is stained for HGF. Likewise, EGF (E) is highly expressed in the stromal regions of the admixed Skov-3 tumor. (F) IL-6 staining is expressed in the Skov-3 alone tumors, however the expression is increased in the admixed tumors.

The third characteristic of TAF is the expression of myofibroblast-like cell markers, including α-SMA, desmin and another marker of neo-microvascularization, VEGF. In our admixed Skov-3/MSC tumors, IHC staining revealed elaborate looped and branched pattern arrangement of both α-SMA and desmin (**Figure 3A and 3B**). VEGF expression was visible in long patches, but not distributed throughout the whole tumor section. These patterns of expression were not evident in the Skov-3–only tumors (**Figure 3C**). The α-SMA staining is an indicator of microvascularization within the tumor and is a contributing factor to the TAF phenotype within the tumor microenvironment.

The fourth and final characteristic of TAF is the expression of growth factors that support tumor development. We confirmed the presence of HGF, EGF and IL-6 within the tumor stimulated MSC population that we identify as TAF. The HGF and EGF staining by IHC were present at concentrated amounts on the leading edge of tumor where a large portion of stroma is found in the admixed Skov-3/MSC tumors as compared to the Skov-3 only tumors (**Figure 3D and 3E**). The IL-6 expression was found in patches and at low levels in the Skov-3 only tumors. In the admixed MSC/Skov-3 tumors, there was a widespread presence of the IL-6 staining that was found in stronger concentrations along the leading edges of the tumor (**Figure 3F**). Growth factors stain in patches that do not correlate with the more sparse staining patterns of the TAF markers themselves due to the fact that the tumor cells also secrete the growth factors HGF, IL-6 and EGF in the presence of MSC as demonstrated by *in vitro* co-culture data (**Figure S4**).

### *In vivo* Skov-3 tumor growth

**Figure 4:**
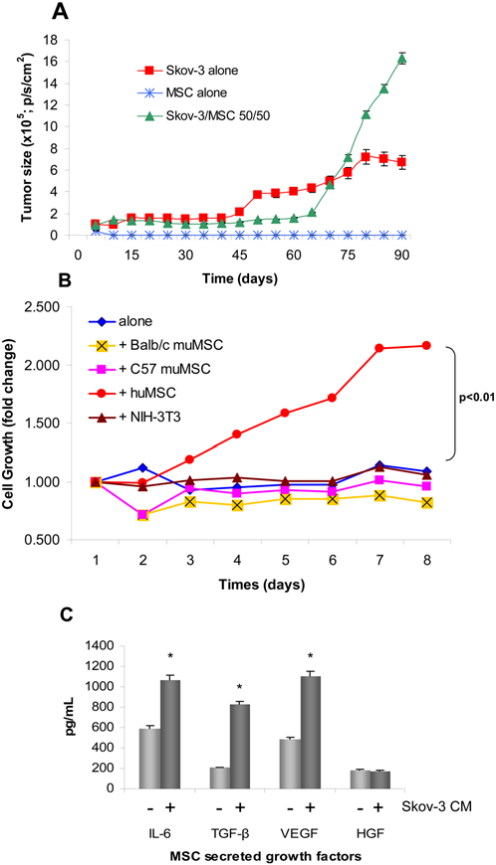
huMSC secreted growth factors promote growth of Skov-3 tumors. The TAF phenotype includes secretion of pro-tumorigenic growth factors. (A) Supported by the secretion of growth factors by the huMSC. *In vivo* growth of xenograft Skov-3 tumors steadily progress but when mixed at a 1∶1 ratio with MSC rapid growth ensued after day 65. At day 91, the Skov-3/MSC 50/50 tumors were significantly larger than the Skov-3 alone (*P*<0.05). (B) Skov-3 tumor cell growth is species dependent. The Skov-3 ovarian tumor cells were growth in co-culture with huMSC, and several muMSC including cells isolated from balb/c and C57 mice, and a fibroblast cell line, 3T3. Growth is graphed as fold change relative to normal Skov-3 proliferation. The only cell line that produced adequate factors to induce cell growth is the huMSC (red circles). The huMSC induced Skov-3 growth significantly (*P*<0.01) better than the other cell lines. (C) Naïve MSC produce basal levels of IL-6, TGF-β, VEGF and HGF. All secreted factors with the exception of HGF are significantly increased upon stimulation with Skov-3 CM (*P*<0.001).

The size of the admixed rLuc labeled-Skov-3/huMSC and rLuc labeled-Skov-3—only tumors was measured by bioluminescent imaging (**Figure 4A**) two times per week until termination of the experiment on day 91. Tumors consisting of Skov-3 cells only reached an average size of 7.05×10^5^ p/s/cm^2^ after steady growth from day 40. However, tumors composed of Skov-3 cells and MSC injected in a 50∶50 ratio displayed a slight relative growth lag until day 80 when rapid growth rates were observed. By day 91, these tumors reached an average size of 1.69×10^6^ p/s/cm^2^, significantly larger than the Skov-3 alone (*P*<0.05). Mice were sacrificed due to excess tumor burden on day 91. This tumor model typically has a 160-day lapse between tumor engraftment and full tumor growth (data not shown). However, the addition of 50/50 Skov-3/MSC shortened the survival of the Skov-3 bearing mice by nearly 70 days. Control mice were sacrificed for IHC comparison to the Skov-3/MSC 50∶50 tumors, but not due to tumor burden. The mice receiving only huMSC did not develop any tumors (**Figure 4A**).

### Skov-3 tumor proliferation is species specific

Species specificity was confirmed by an *in vitro* tumor growth assay (TGA) using admixed human ovarian Skov-3 cells and murine MSC (muMSC) in the same 1∶1 ratio used previously to determine whether the murine cells had any significant effect on the xenograft. The Skov-3 cells were mixed with muMSC derived from Balb/c or C57 mice, 3T3 murine fibroblasts, or huMSC. Cell proliferation was graphed as fold change compared to Skov-3 tumor cell proliferation alone. The murine stromal lines did not significantly hinder the Skov-3 cell proliferation. The Balb/c MSC reduced proliferation by a 0.75 fold difference, C57 MSC by a 0.94 fold difference and NIH-3T3 by a 1.1 fold differnence. However, the huMSC admixed significantly increased Skov-3 cell proliferation by 2.25 fold difference (*P*<0.01) after 8 days of co-culture (**Figure 4B**).

### MSC secreted growth factors support Skov-3 cell proliferation

Growth factors that support tumor cell growth are secreted by both MSC and the tumor cells when reciprocally stimulated by one another. An initial ELISA array of several growth factors secreted by MSC shows a significantly increased secretion (*P*<0.001) of TGF-β, VEGF and IL-6 following 24 hour culture with Skov-3 conditioned medium (CM), while HGF secretion remains steadily expressed (**Figure 4C**). The reciprocal regulation of MSC conditioning on stimulated Skov-3 tumor showed increases in IL-6, TGF-β, VEGF and HGF secretion (**Figure S4**).

### MSC contribution to tumor growth

**Figure 5:**
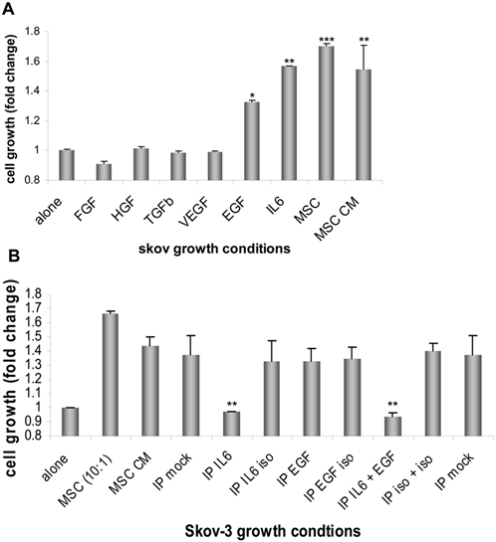
Growth factors are critical to Skov-3 tumor progression. (A) An *in vitro* 3D tumor growth assay (TGA) at day 8. Briefly, RFP-labeled Skov-3 tumor cells were mixed with huMSC cells, huMSC conditioned media (CM) or various recombinant cytokines (FGF, HGF, TGF-β, VEGF, EGF, IL-6) in a 3D assays (as described in the materials and methods). EGF (*P*<0.05) and IL6 (*P*<0.01) significantly increased the proliferation of Skov-3 cells compared to the Skov-3 only, but less than the MSC (*P*<0.01) induced Skov-3 proliferation. (B) Immunoprecipitation of IL-6 from the Skov-3/MSC co-culture medium over an 8 day period reveals significantly reduced growth of the Skov-3 (*P*<0.01) cells as compared with the Skov-3/MSCco-culture. Cell growth is graphed as fold change relative to normal Skov-3 proliferation.

Because we confirmed that enhancement of growth in our xenograft tumor model was specific to factors produced by huMSC and not by the mouse itself, we conducted an *in vitro* TGA to determine rLuc/RFP labeled Skov-3 cell proliferation over an 8-day period under multiple co-culture conditions. Proliferation was graphed in terms of fold change relative to Skov-3 alone. Exposure to huMSC (*P*<0.01) or huMSC-conditioned medium (*P*<0.01) significantly increased growth of Skov-3 cells (**Figure 5A**), suggesting that growth promotion by MSC is mediated by a paracrine effect. MSC can secrete growth factors to support tumor cell growth. MSC co-cultured with Skov-3 cells of Skov-3 CM secrete growth factors such as HGF, IL-6, TGF-β, VEGF, bFGF and EGF. Each of these factors were added individually as recombinant proteins to the Skov-3 cell TGA culture. The HGF, bFGF, TGF-β and VEGF had minimal impact on Skov-3 proliferation, while added EGF (*P*<0.05) and IL-6 significantly (*P*<0.01) enhanced Skov-3 growth rates (**Figure 5A**). Skov-3 tumor proliferation was measured based on fold change compared to normal Skov-3 cell proliferation in low serum media as detailed in the methods section.

To confirm the importance of huMSC-secreted EGF and IL-6 on Skov-3 cell proliferation, we co-cultured huMSC and rLuc/RFP labeled Skov-3 *in vitro* and immunoprecipitated (IP) EGF and/or IL-6 out of solution (individually and in combination). IP of IL-6 from the MSC-CM showed significantly decreased Skov-3 proliferation (*P*<0.01; **Figure 5B**). IP of EGF from the MSC-CM was not significant and showed similar Skov-3 proliferation to that of the isotype controls. While several growth factors may be necessary for the *in vivo* progression of the Skov-3 tumor, *in vitro* studies revealed huMSC-induced IL-6 secretion to be critical for the enhanced proliferation observed in Skov-3/MSC TGA.

## Discussion

Tumor stroma, fibrovascular networks and the tumor microenvironment are vital to the structural integrity of the tumor and its preservation/proliferation. TAF cells are derived from cells of two different origins, most likely a combination of both. The first is the bone marrow-derived progenitor population of which both hematopoietic stem cells [6] and MSC [12], [21], [22] are suggested as precursors; the second is a tissue-resident MSC population. MSC are not only a bone marrow resident population of cells, but they are also a tissue resident population and may even be a circulating population. In our experiments we set out to show that MSC, in toto, is the precursor to the tumor associated fibroblast (TAF).

TAF are defined as an activated fibroblast population, which is a rich source of growth factors, such as HGF and TGFβ [10]. Activated fibroblasts form fibrovascular stromal patterns, a diverse arrangement of septa within tumors; their presence represents disordered, rapid tumor growth and can be associated with heightened tumor aggressiveness and poor prognostic outcome [23]. We show the “activation” of MSC by tumor conditioned media by the increase of TAF-like surface markers (**Figure 1A**). Activated fibroblasts and myofibroblasts contribute to the formation of microvascular structures and can be detected by α-SMA and desmin expression [24], [25]. Recently, published data identify pericyte characteristics by α-SMA staining within an *in vivo* glioma tumor model of systemically injected MSC [26]. MSC are pericytic precursors [27], and thus we hypothesize that they are precursors of TAF, which are identified by pericytic, myofibroblastic and “activated” fibroblastic markers. These TAF arise from the admixed MSC population to promote growth, matrix remodeling, and neovascularization of xenograft tumors.

MSC represent a relatively well-defined *ex vivo* cell population with complex and poorly understood biology *in vivo* [28], [29]. MSC-based anticancer therapies represent an exciting and novel therapeutic approach, but a better understanding of how MSC home to, engraft within and ultimately have an impact on tumor behavior will be essential before we can realize the full potential including the risks of using of MSC-based delivery systems in a clinical setting. The first indication that MSC had a natural affinity for tumors was reported by our group in 2001 [30]. In those and subsequent studies, we demonstrated that MSC, administered intravenously, were able to integrate and persist within tumor stroma of pre-established human melanoma, breast cancer and glioma xenografts [11], [12], [16]. These data were confirmed in other tumor models [31], [32], most recently by Weinberg *et al.* [33]. We also demonstrated that MSC could deliver therapeutic doses of interferon-beta, locally within a tumor, and demonstrated that this treatment had significant anti-tumor effects [11], [12], [16]. The use of cell-based delivery systems such as MSC holds clinical promise and significant current research effort is focused in this area. We provide evidence herein, that the MSC has the potential to be tumor supportive. This should not dissuade the pursuit of MSC as gene delivery vehicles, but instead, serve as a caveat in the preemptive strike against potential pitfalls in the future of targeted gene delivery.

In these experiments, we show enhanced tumor growth is the effect of a 1∶1 ratio of tumor:MSC *in vivo*. However, in previous studies with altered tumor:MSC ratios, MSC have had adverse effects on tumor growth, prolonging the survival of the mice by hampering tumor proliferation (data not shown) [34]–[37]. In contrast to MSC inhibiting tumor progression and similar to what is described within this paper, our previous data shows that MSC can enhance the growth rate of a subpopulation of breast cancer cells *in vitro* and *in vivo* through paracrine interleukin-6 (IL-6) signaling events [38], [39].

Through IHC, we initially observed the formation of a fibrovascular structure and “TAF characteristics” by systemically circulating MSC engrafting within established xenograft tumor models. Figure S3 shows the expression of the TAF markers: α-SMA, desmin, Tn-C and TSP-1 in ovarian, breast and pancreatic tumor models. Each of these tumor models received injections of MSC, yet after 14 weeks, the participation of the MSC in the tumors resulted in the expression of the same TAF markers observed in the admixed Skov-3/MSC tumor model. Interestingly, although each tumor type utilized the MSC as a TAF, as confirmed by the presence of α-SMA, desmin, Tn-C and TSP1, similarities between the IHC staining patterns do not exist across tumor types. Potentially due to the heterogeneity in paracrine factor expression between breast, ovarian and pancreatic tumor types, each tumor appears to elicit different responses or requirements from MSC, however, the presence of the TAF-markers is a unifying factor between the all three tumors.

Subsequently, an admixed xenograft experiment of Skov-3 ovarian tumor cells and huMSC was used to further demonstrate the formation of fibrovascular networks in the admixed Skov-3/MSC tumors and not in the Skov-3-only tumors, indicating that MSC give rise to TAF within the tumor microenvironment. First, the four defining characteristics illustrated in Figure 1 are sought by IHC staining of 91-day-old admixed Skov-3/MSC xenograft tumors. We indicate the neovascular, fibroblastic, pericytic, and matrix remodeling nature of the TAF as well as the overall contribution of the TAF to the leading edge stromal component of the tumor microenvironment.

The first characteristic requires expression of two fibroblastic markers: FAP, which is expressed on MSC and fibroblasts [40] and FSP, a marker overexpressed in TAF as compared to normal fibroblasts [41]. The expression of both markers is evident in both the systemic-MSC injected model and the admixed MSC model and furthermore not evident in the Skov-3-only IHC staining (**Figure 2A and 2B** **, Figure S3**).

The second characteristic is the degree of expression of the proteins of “aggression” such as SL-1, otherwise known as MMP-3, a degradative enzyme highly expressed by fibroblasts [42] and utilized in tissue remodeling by TAF [21]. Positive staining for SL-1 was identified in Skov-3/MSC admixed tumors but Skov-3 alone as were other pathogenic-associated glycoproteins Tn-C and Tsp-1 (**Figure 2C, 2D, and 2E**).

The third characteristic is the expression of typical myofibroblast-associated proteins. Desmin, a muscle-specific, intermediate filament protein common in myofibrils [43], was expressed in huMSC derived fibroblasts in a heterogeneous pattern of distribution. The expression patterns of desmin are comparable to the global expression pattern observed on staining for α-SMA of admixed Skov-3/MSC tumors (**Figure 3A and 3B**). α-SMA positivity has been used previously to identify differentiated MSC [44]. Our data suggests that MSC do contribute to the perivascular formation within the admixed Skov-3 tumors because much of the human specific α-SMA and desmin positive staining is evident around tumor vessel structures.

Typically, the cells associated with the abnormal, tumor-associated, fibrovascular septa are not the archetypal vascular endothelial cells classified by factor VIII, CD31 and CD34, but instead are networks consisting of collagen and laminin subtypes in addition to activated fibroblasts and macrophages [45]. Initial gene-expression profiles of long-term Skov-3—conditioned MSC reveal the upregulation of several laminin and collagen subtypes (data not shown); the deposition of these matrix proteins is associated with pre-vasculogenesis in preparation for the later stages including angiogenesis [46]. The TAF potential to support microvascular sprouting corroborates the *in vivo* tumor growth advantage of the admixed Skov-3/MSC tumors (**Figure 4A**). The microvascular formation that we elude to is restricted *in vivo* tumor growth (depicted in cartoon fashion in **Figure S2**) and is characterized by networks of straight, looping and branching arced speta seen in both the α-SMA and desmin stained slides (**Figure 3A and 3B**). Abnormal septa formation is indicative of vascular support due to the presence of the MSC (TAF) within the tumor microenvironment and is otherwise not observed. Microvascular channel formation is specific to tumor vasculogenesis and is featured by the small, un-orderly arrangement of microvessels that we depict through α-SMA, desmin and VEGF staining (**Figure 3**).

The fourth and final characteristic that defines a TAF is secretion of tumor-supportive growth factors, including HGF, EGF and IL6 (**Figure 3D, 3E, and 3F**). The growth factors secreted by the MSC are known to induce a TAF-like phenotype, thus providing autocrine stimulation to the MSC itself to differentiate into a TAF. Skov-3 cells and MSC interact in a paracrine manner by inducing reciprocal growth factor expression including IL-6, VEGF, HGF and TGF-β (**Figure 4C** **and Figure S4**). TGF-β induces HGF expression by fibroblasts [47] but also induces the transition of fibroblasts to myofibroblasts by increasing α-SMA and Tn-C expression [48]. Several adenocarcinoma tumor-microenvironment crosstalk models implicate growth factors including HGF, [49] VEGF, [50] TGF-β, and FGF [51] in the promotion/progression of tumorigenesis. The HGF/c-Met interaction plays a pivotal role in cancer invasion that is mediated by the tumor stromal cell production of HGF [52], which can induce ECM degradation, tubule formation and angiogenesis [53]. HGF also has the ability to transactivate the EGF receptor to induce motility [54] and reciprocally, EGF is able to stimulate HGF production [52].

In our *in vitro* studies, both MSC and Skov-3 cells showed reciprocal induction of growth factors (IL-6, TGF-β1, VEGF and HGF) following 24 hours conditioning (**Figure 4C** **and Figure S4**). Only EGF and IL-6 and not FGF, HGF, TGF-β1 or VEGF proved to be essential components of the growth advantage observed for the mixed labeled Skov-3 TGA cultures in comparison with the labeled-Skov-3-only cultures for the first 8 days of co-culture. The *in vitro* presence of MSC, MSC-conditioned medium EGF and IL-6 is essential to the growth of tumor cells in a controlled Matrigel environment without the presence of MSC (**Figure 5A**). Furthermore, MSC-conditioned medium stripped of IL-6 induced the same growth rates observed in the Skov-3 tumor cells alone (**Figure 5B**). While recombinant EGF enhanced Skov-3 proliferation (**Figure 5A**), the immunoprecipitation of EGF from MSC conditioned medium did not significantly effect Skov-3 tumor growth. MSC-secreted EGF, *in vitro,* is not sufficient to enhance Skov-3 proliferation as seen with MSC-secreted IL-6. On the basis of our *in vitro* tumor growth assay data, we conclude that the MSC produced paracrine factors HGF, FGF, TGF-β, VEGF and EGF do not individually affect the growth rate of the Skov-3 xenograft, whereas IL-6 does enhance the proliferation as confirmed by the IP of IL-6 out of MSC conditioned medium (**Figure 5B**).

The discrepancy between the *in vivo* and *in vitro* data is significant, however, the *in vitro* data measured the first 8 days of tumor cell proliferation, whereas the *in vivo* data revealed day 91 of tumor proliferation. Accordingly, we suggest that several growth factors mediate the transition from the tumor supportive MSC to the tumor supportive TAF. *In vitro,* only IL-6 is significant to the initial ovarian cell line proliferation. While none of the other factors showed significant effects on tumor cell proliferation individually, the significance and contribution of these factors observed in the xenograft model should not be overlooked. The presence of HGF and EGF *in vivo* (**Figure 3D and 3E**), may have an impact on growth later phase growth (past day 8) or likewise in other tumor models.

Like estrogen receptor alpha (ERα)—positive breast cancer cells, on which we have previously reported [38], [39], our MSC-enhanced Skov-3 growth relies on MSC-secreted paracrine factors, such as IL-6, and not strictly on cell-cell contact. Unlike MSC-supported ERα-positive breast cancer cells, however, Skov-3 ovarian cancer cell growth is enhanced by IL-6. This exemplifies the diversity of the MSC as a functionally supportive cell in multiple environments. We showed here that MSC secreted IL-6 is key to the growth of the Skov-3 ovarian tumor model while we showed previously the importance of MSC-secreted EGF to the ERα-positive breast cancer model. MSC are able to respond to distinctive stimuli from individual microenvironments to elicit the proper function that environment.

In conclusion, the MSC play an integral role in the development of the mature, complex, physiological tumor microenvironment. This study represents an early step in better defining the biological activities of MSC within said environment. Our finding that MSC secreted paracrine factor, IL-6, enhances early stage Skov-3 proliferation emphasizes the importance of a proper microenvironment for tumor growth. Furthermore, we have shown that the MSC is an essential component within the tumor microenvironment, as it contributed to fibrovascular formation within that microenvironment that were not evident in Skov-3 only tumors. The tumor microenvironment within a xenograft mouse model occupied by MSC becomes a much more intricate environment, mimicking the complex physiological tumor microenvironment in humans. Reiteration of the diverse phenotype of the TAF is necessary; TAF can be matrix-synthesizing or matrix-degrading cells, they can be contractile cells (myofibroblasts), circulating precursor cells (fibrocytes), or blood vessel-associated pericytes [10], [21]. Nonetheless, TAF, in whichever form they represent in the tumor microenvironment, biologically impact tumor progression through the production of growth factors, cytokines, chemokines, matrix-degrading enzymes, and immunomodulatory mechanisms.

## Materials and Methods

### Cell isolation and culture

Normal huMSC were provided through the Tulane Center for Gene Therapy, MSC cell distribution center (Darwin Prockop, Tulane University, New Orleans, LA). Tulane Center for Gene Therapy provides well-characterized human mesenchymal stem cells. These adult stem cells are the plastic adherent fraction from bone marrow. The cells are isolated, expanded and extensively characterized (see URL link for characteristics of the cells, including phenotypic analysis, proliferation rate, expansion data, colony forming units, and trilineage differentiation into mineralizing cells, adipocytes and chondrocytes: http://www.som.tulane.edu/gene_therapy/distribute_docs/Spec_Sheet.pdf). These cells were thawed, and expanded in alpha-minimum essential medium (MEM; Mediatech, Herndon, VA) 20% fetal bovine serum (FBS; Invitrogen, Carlsbad, CA) at 37°C. After 1 passage cells were split and a fraction of the cells were phenotypically analyzed to ensure the quality of the cells. MSC were expanded passage number 1–4 were used in the subsequent experiments.

Human ovarian cancer Skov-3, breast cancer MDA-231 and pancreatic cancer Panc-1 cells were obtained from American Type Culture Collection (ATCC, Manassas, VA) and cultured in minimum essential medium/Earl's salts non-essential amino acid medium supplemented with 10% FBS, L-glutamine and the penicillin-streptomycin mixture (Invitrogen, Carlsbad, CA). Murine MSC (mMSC) cell lines from C57/B6, balb/C were isolated as described previously [55] Fibroblast 3T3 cells and mMSC were cultured in alpha-MEM containing 20% FBS and 10% LPS. Skov-3 tumor cells were stably transduced using a lentivirus expressing red fluorescent protein and renilla luciferase (rLuc) [56], [57].

### Animals

Female CB-17 SCID mice were purchased from Harlan (Indianapolis, IN). Mice were housed and used in accordance with institutional guidelines of the University of Texas, M.D Anderson Cancer Center under IACUC approved protocols (FC Marini). The UTMDACC's animal care and use program has been fully accredited by the Association for the Assessment and Accreditation of Laboratory Animal Care International (AAALAC).

### Xenograft mouse models

#### Admixed MSC/Skov-3

The admixture (co-injection) studies were carried out with 4×10^6^ cells comprising Skov-3 and huMSC cells in the following ratios: huMSC alone (n = 5), Skov-3 alone (n = 5), or 50∶50 mixture (n = 5). All injections were given to anesthetized animals; tumor cells were suspended in 200 µl PBS and administered via subcutaneous administration to the shaven rear flank. Mice were observed and tumor growth was measure by both bioluminescent imaging (Xenogen IVIS bioluminescence/fluorescence optical imaging system; Caliper Life Sciences, Hopkinton, MA) until day 91 when mice were euthanized due to excess tumor burden. Tumors were removed and stored in OTC compound (Miles, Inc., Elkhart, IN) and then snap-frozen in liquid nitrogen and stored at −80°C until tissue processing.

#### Systemic MSC Injection

Mice received an intraperitoneal injection of 4×10^6^ Skov-3 cells suspended in 1 ml PBS on day zero. MDA-231-only and Panc-1-only xenograft mice received a subcutaneous injection of 1×10^6^ cells in the mammary fat pad or lateral flank, respectively. Survival was measured from the day of tumor cell injection until the day of death. Tumor engraftment was evident by palpable tumor after 12 days. For each of the experiments, fifteen of these mice received four weekly injections, 2 weeks post tumor engraftment, of 1×10^6^ huMSC alone (1 ml PBS suspension ip for the Skov-3 model or 100 µl PBS suspension iv for the breast and pancreatic models); a control group (n = 4) received no huMSC. Mice were euthanized on week 14. Tumor growth was measured biweekly using bioluminescent imaging (IVIS-Xenogen 200; Caliper Lifesciences, Hopkinton, MA) end-point tumor size was measured with a caliper as well as in vivo imaging. Tumors were removed and stored in OTC compound (Miles, Inc., Elkhart, IN) and then snap-frozen in liquid nitrogen and stored at −80°C until tissue processing.

### Tissue processing

On day 91, all mice were sacrificed and tumor tissues were removed and embedded in OTC compound (Miles, Inc., Elkhart, IN), snap-frozen in liquid nitrogen and stored at −80°C. Frozen tissue was sectioned (6–8 µm) and processed for hematoxylin-eosin or IHC staining. Sections were imaged with a Zeiss Axioplan2 microscope (Carl Zeiss Inc, Thornwood, NY) equipped with a charge-coupled device (CCD) camera (Hamamatsu Co., Bridgewater, NJ) and Adobe Photoshop software (Adobe Systems Inc., San Jose, CA).

### Imaging studies

Tumor growth was followed through noninvasive *in vivo* optical imaging (Xenogen IVIS bioluminescence/fluorescence optical imaging system; Caliper Life Sciences, Hopkinton, MA) biweekly for 91 days. Five minutes prior to imaging, each mouse was given an IP injection of a 100 µl injection of 40 mg/ml coelenterazine as described previously [56]. General anesthesia was then induced with 5% isoflurane (IsoSol, Medeva Pharmaceutical PA, Inc.); the mouse was placed in the light-tight heated chamber during which anesthesia was continued with 2% isoflurane introduced via nose cone. The imaging system consists of a cooled, back-thinned CCD camera to capture both a visible light photograph of the animal taken with light-emitting diodes and the luminescent image. Luminescent images were acquired with 1- to 3-minute exposure times. Optical images were displayed and analyzed with IVIS Living Image (Caliper Life Sciences [Xenogen], Hopkinton, MA) software packages. Regions of interest were manually drawn around the bodies of the mice to assess signal intensity emitted. Optical signal was expressed as photon flux, in units of photons/second/centimeter^2^ (p/s/cm^2^). Tumor growth is translated into photon flux as a unit of measurement directly proportional to the number of rLuc-expressing tumor cells.

### Immunohistochemical analysis

All antibodies were human specific and were exhaustively tested against multiple mouse tissues to confirm the species specificity and non–cross-reactivity between species. huMSC differentiation *in vivo* was examined in frozen sections of mouse tumor xenografts on day 91 of tumor growth when animals were sacrificed. Adjacent tumor sections were only used to show MSC co-localization with α-SMA; all other sections presented are not directly adjacent to one another. Sections were fixed for 5 minutes in neutral buffered formalin, after which endogenous peroxidase activity was quenched by incubating the sections in 0.3% hydrogen peroxide in methanol for 30 minutes. The sections were treated with the following antibodies: rabbit anti-human FSP, mouse anti-human FAP and mouse anti-human Tn-C (1∶1000 dilution; Abcam, Cambridge, MA), goat anti-human HGF (1∶1000 dilution; R&D Systems, Minneapolis, MN) and mouse anti-human thrombospondin (1∶1000 dilution; Lifespan Biosciences; Seattle, WA), mouse anti-human α-SMA (1∶1000 dilution; Biomeda, Foster City, CA), rabbit anti-human desmin (diluted 1∶1000; Novus Biologicals, Littleton, CO), mouse anti-human Thy 1 (BioLegend, SanDiego, CA), mouse-anti-human SL-1, mouse anti-human VEGF, mouse anti-human EGF, (1∶1000 dilution; Santa Cruz Biotechnology Inc, Santa Cruz, CA), mouse antistromal antigen (1∶1000 dilution; Dianova, Hamburg, Germany), rat anti-human IL-6 (1∶200; Abcam, Cambridge, MA) and mouse anti-human TGF-βIIR (1∶1000; DakoCytomation, Carpinteria, CA). We followed the manufacturer's procedures for the goat, rabbit or mouse peroxidase kits (Vector Laboratories, Burlingame, CA). Peroxidase substrate was developed by using the AEC (3-amino-9-ethylcarbazole) and/or DAB (3,3′-diaminobenzidine) substrate kit (Vector Laboratories). Slides were counterstained with hematoxylin QS (Vector Laboratories) and were either mounted with low-viscosity aqueous mounting medium (Scytek Laboratories, Logan, UT) or dehydrated and mounted with VectaMount Permanent Mounting Medium (Vector Laboratories).

### *In vitro* tumor conditioning

MSC were grown to 40% confluency in α-MEM supplemented with 20% FBS and 10% PSG in T-175 flasks. Skov-3 cells were cultured to 50% confluency before they were switched to a serum free growth medium. After 2 days, the Skov-3 medium was removed, filtered (0.2 µm filter) and placed on the MSC. MSC were conditioned for 24 hours prior to ELISA analysis of TGF-β, IL-6, VEGF and HGF. Reciprocal 24 hour conditioning was carried out for the MSC conditioned Skov-3 tumor cells prior to ELISA measurements of TGF-β, VEGF, HGF and IL-6. The long term conditioned MSC were conditioned with Skov-3-conditioned medium for 16 days. Media was changed every 3 days and MSC were maintained between 40% and 80% confluency. Cells were then analyzed by western blot for the presence of TAF-like protein expression. Controls cells for ELISA and western blot were conditioned in serum free α-MEM.

### Western blot

MSC, fibroblasts, and Skov-3 cells were grown and conditioned on 10cm tissue culture dishes. When cells were 50% confluent, they were serum starved overnight before 16 day-conditioning. Cells were maintained at a cell density of 4000 cells/cm^2^. After 16 days, cells were washed 2× with PBS, scraped in PBS and spun down at 300 rpm for 5 minutes before the addition of 50 µL of phosphoprotein (NP40) lysis buffer (25 mm HEPES, 150 mm NaCl, 1 mm EDTA, 25 mm NaF, 5% glycerol, 1 mm Na-orthovandate, 1% NP40, 0.5% Triton x-100, 1.4 mg/ml aprotinin, 100mm PMSF, 250 mg/mL benzamindine and 1.25 M β-glycerol phosphate). Lysates were separated on a 12% polyacrylamide gel, transferred to Hybond-P membranes (Amersham Pharmacia Biotech, Buckinghamshire, England) and probed with primary antibodies. Cell lysates were added at 20 µg per well and run for 90 minutes at 100 volts. Transfers were performed for 2.5 hours at 400 mA. Antibodies used for western include, FAP (1∶200 dilution; Abcam, Cambridge, MA), FSP (1∶200 dilution; Lifespan Biosciences; Seattle, WA), desmin (1∶200 dilution; Abcam, Cambridge, MA), Tsp-1 (1∶400 dilution; Lifespan Biosciences; Seattle, WA), α-SMA (1∶200 dilution; Abcam, Cambridge, MA), Tn-C (1∶200 dilution; Abcam, Cambridge, MA), tubulin (1∶2000 dilution; Cell Signaling), secondary anti-mouse and anti-rabbit antibodies (1∶1000 dilution; Cell Signalling) Bands were visualized using an enhanced chemiluminescence plus kit (Amersham Pharmacia Biotech).

### Flow cytometry

MSC that reached 80% confluency were trypsinized and washed three times with PBS, then 1×10^5^ cells were resuspended in 1 µg/ml of primary antibody or conjugated antibody for 15 minutes on ice. Cells were then washed two times in PBS and resuspended in a 1∶25 dilution of anti-mouse-phycoerythrin (PE)-secondary antibody for 15 minutes on ice. Cells were washed two times in PBS and resuspended in 250 µl PBS on ice until analyzed on an LSRII flow cytometer (BD Biosciences, San Jose, CA). Unconjugated antibodies used included desmin, α-SMA, Tn-C, FAP, FSP (Abcam, Cambridge, MA) with anti-mouse or anti-rabbit PE secondary (Abcam, Cambridge, MA), Conjugated antibodies utilized included PE-conjugated CD105, CD90, CD73, CD44, CD31, CD34 and CD45 (BD Biosciences, San Jose, CA).

### Tumor growth assay (3D TGA)

*In vitro* tumor cell proliferation is detected by the fluorescence-based 3D TGA that noninvasively monitors tumor cell growth for eight days after cells are embedded in Cultrex BME at 3 mg/mL [38], [39]. Briefly, red fluorescent (rLUC/RFP-labeled) Skov-3 tumor cells were embedded (in triplicate) in a 100 µL plug of 3 mg/mL Cultrex BME in a black-walled, clear-bottom 96-well plate at 12,500 tumor cells (±) 3D MSC-CM, 3D MSC-CM(-GF) (i.e., 3D MSC-CM with one of several growth factor (GF) listed below removed via immunoprecipitation), or recombinant GF as indicated. Wells were overlaid with phenol red–free, serum-free RPMI 1640. Individual well fluorescence intensities were monitored daily for eight days. Relative Skov-3 growth rates were documented as relative fluorescence units and graphed as fold change (growth). An all inclusive description of this assay and its utility for evaluating growth rate kinetics of breast cancer cells in complex 3D tumor-like microenvironments can be found in our previously published studies [38], [39].

#### Soluble protein quantification

MSC-CM was assayed for IL-6, EGF, HGF, VEGF, TGF-β1 and FGF protein levels using the respective DuoSet Human ELISA (R&D Systems). The ELISA was performed according to the manufacturer's instructions. In addition, each growth factor was removed from a subset of MSC-CM from 3D/Cultrex BME by GF immunoprecipitation (see below).

#### GF immunoprecipitation

GF (IL-6, EGF, HGF, VEGF, TGF-β1 and FGF) was immunoprecipitated as previously described [39]. The 3D MSC-CM was incubated with 2 µg/mL anti-GF monoclonal antibody (Mab206, Mab636, Mab294, Mab293, Mab1835 and AF233NA; R&D Systems) for 4 to 6 h at 4°C with constant rotation. To verify that each GF had been removed from the conditioned medium, an aliquot of conditioned medium stripped of GF via immunoprecipitation was tested for the respective GF concentration by ELISA, and in all cases, GF concentrations were >14-fold less as determined by ELISA.

## Supporting Information

Figure S1 MSC characterization. MSC were sorted by flow and were positive for CD105, CD90, CD44, CD73, and CD140b, and negative for the endothelial cell marker and hematopoietic markers CD31, CD34, and CD45.
(0.21 MB TIF)

Figure S2 Archetypical stromal patterns found in tumors. The microvascular patterns often found in tumor masses include enlarged vessel walls and septa that are not uniform, but are often arched or branched or looped in pattern, thus creating an unorganized support structure and inefficient vascularization throughout the tumor. The cartoon depiction shows the microvascular structure as stained by α-SMA.
(5.26 MB TIF)

Figure S3 In ovarian, breast, and pancreatic xenograft mouse models, tumors show patterns of fibrovascular networks following intravenous injection of huMSC. Tumor cells, were allowed to engraft prior to administration of huMSC [4 times, once per week, Skov-3 (IP), MDA-231 (IV) or Panc-1 (IV)]. On week 14, mice were sacrificed and tumors for histology were collected. (A) We stained for the presence of MSC (Thy-1) which we showed co-localized expression with α-SMA on adjacent tissue sections. (B) Further tissue sections are not adjacent sections. All three tumor types show fibrovascular network pattern staining of α-SMA, (C) desmin, (D) Tn-C, and (E) TSP-1 within the tumors that were treated with MSC while tumors not treated with MSC showed no staining (negative tumor, Skov-3-only, is representative of MDA and Panc-1 tumors).
(6.51 MB PDF)

Figure S4 Skov-3 tumor cell secretion of growth factors following the stimulation with MSC-CM. Secreted proteins are measured from Skov-3 tumor cell cultures prior to and post stimulation with MSC-CM. IL-6 (P<0.0001), VEGF (P<0.01), HGF (P<0.0001) and TGF-β (P<0.001) secretion are all increased following conditioning with MSC CM.
(0.34 MB TIF)
