# Correction: Mesenchymal Stem Cell Transition to Tumor-Associated Fibroblasts Contributes to Fibrovascular Network Expansion and Tumor Progression

**DOI:** 10.1371/annotation/4ab4c130-16cb-41f0-9507-b00ce070fbc6

**Published:** 2013-03-19

**Authors:** Erika L. Spaeth, Jennifer L. Dembinski, A. Kate Sasser, Keri Watson, Ann Klopp, Brett Hall, Michael Andreeff, Frank Marini

Figure 1A in the published article was composed from different blots. We are making the original films from which the blot was assembled available with this correction (http://www.plosone.org/corrections/pone.0004992.g001.1.cn.tif).

We are also providing a revised Figure 1A composed of four blots that were run on the same gel and detected using fluorescence instead of chemoluminescence (http://www.plosone.org/corrections/pone.0004992.g001.2.cn.tif). The full blots are also included with the figure. Small differences between the Figure 1A included in the original article and the new Figure 1A can be attributed to the different detection method (chemoluminescent vs. fluorescent), different MSC patient sample number and storage conditions (fresh vs. 4+ years at -20°C). However, the trends and the implication of the data as reported in the article remain the same.

