# Supplementary figures and images for: Mesenchymal Stem Cell Transition to Tumor-Associated Fibroblasts Contributes to Fibrovascular Network Expansion and Tumor Progression
Source: PLoS One. 2009 Apr 7;4(4):e4992. doi: 10.1371/journal.pone.0004992 (PMC2661372; doi:10.1371/journal.pone.0004992)

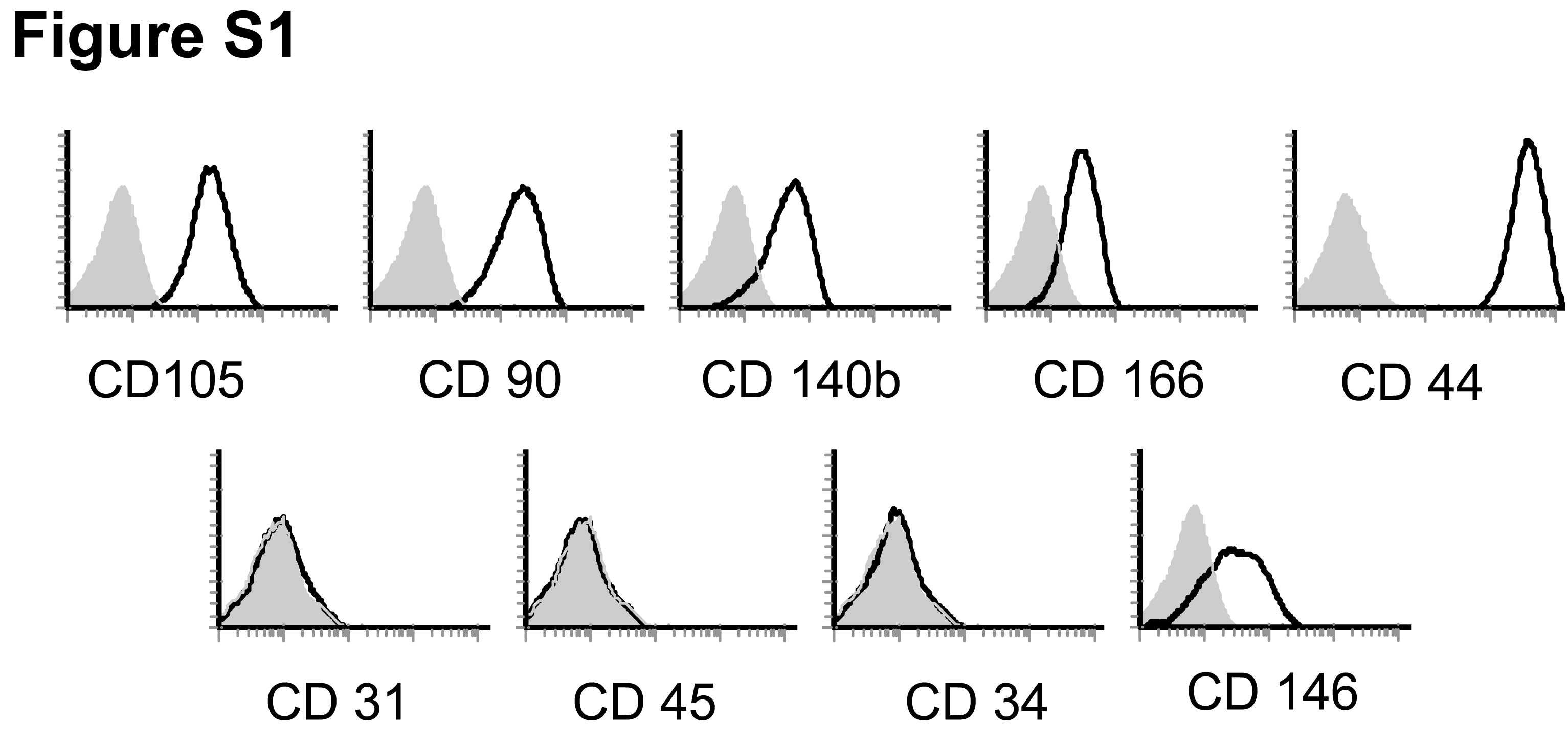

Supplement: Figure S1 — MSC characterization. MSC were sorted by flow and were positive for CD105, CD90, CD44, CD73, and CD140b, and negative for the endothelial cell marker and hematopoietic markers CD31, CD34, and CD45. (0.21 MB TIF) [file pone.0004992.s001.tif]

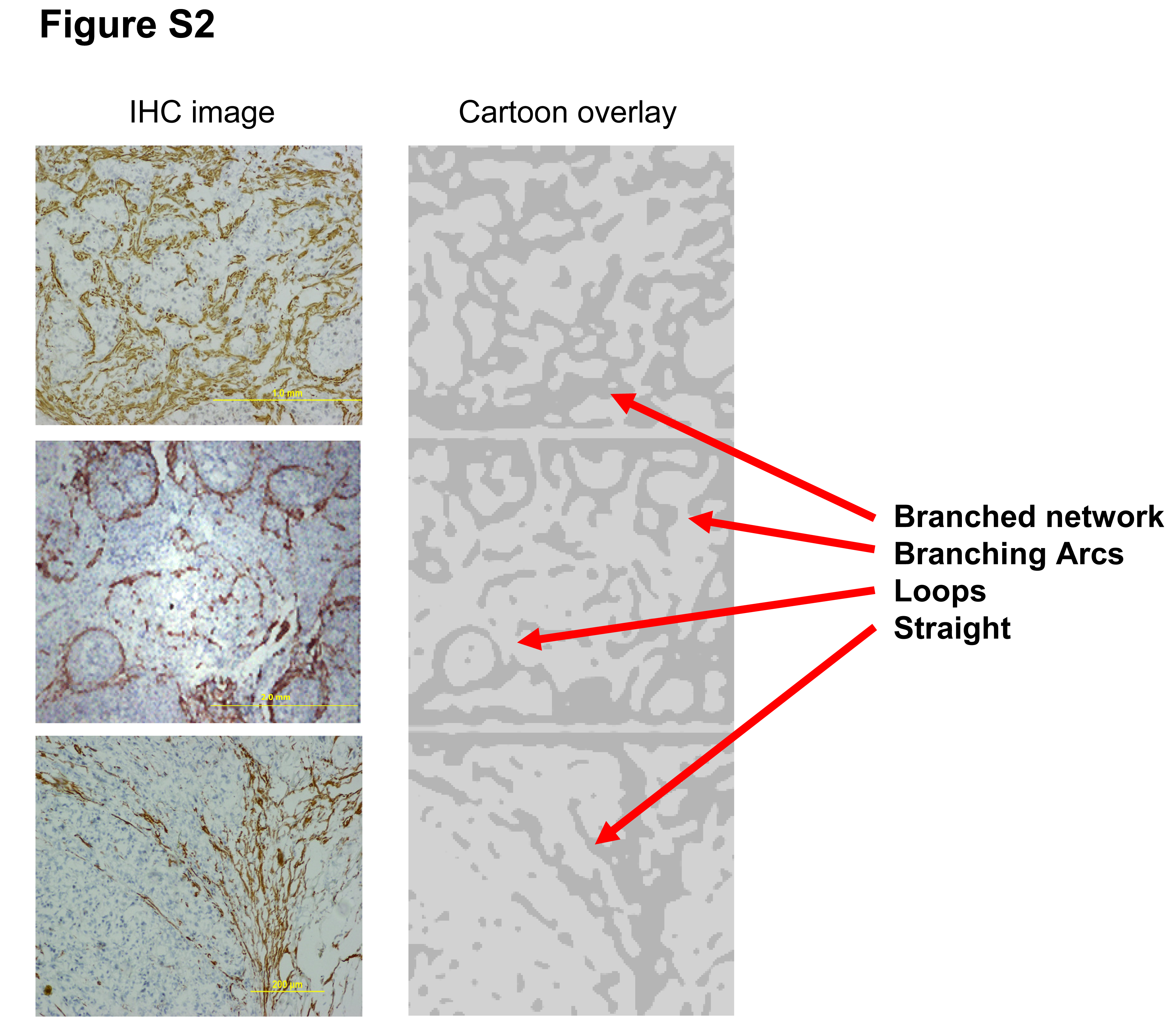

Supplement: Figure S2 — Archetypical stromal patterns found in tumors. The microvascular patterns often found in tumor masses include enlarged vessel walls and septa that are not uniform, but are often arched or branched or looped in pattern, thus creating an unorganized support structure and inefficient vascularization throughout the tumor. The cartoon depiction shows the microvascular structure as stained by α-SMA. (5.26 MB TIF) [file pone.0004992.s002.tif]

Supplemental Figure 3

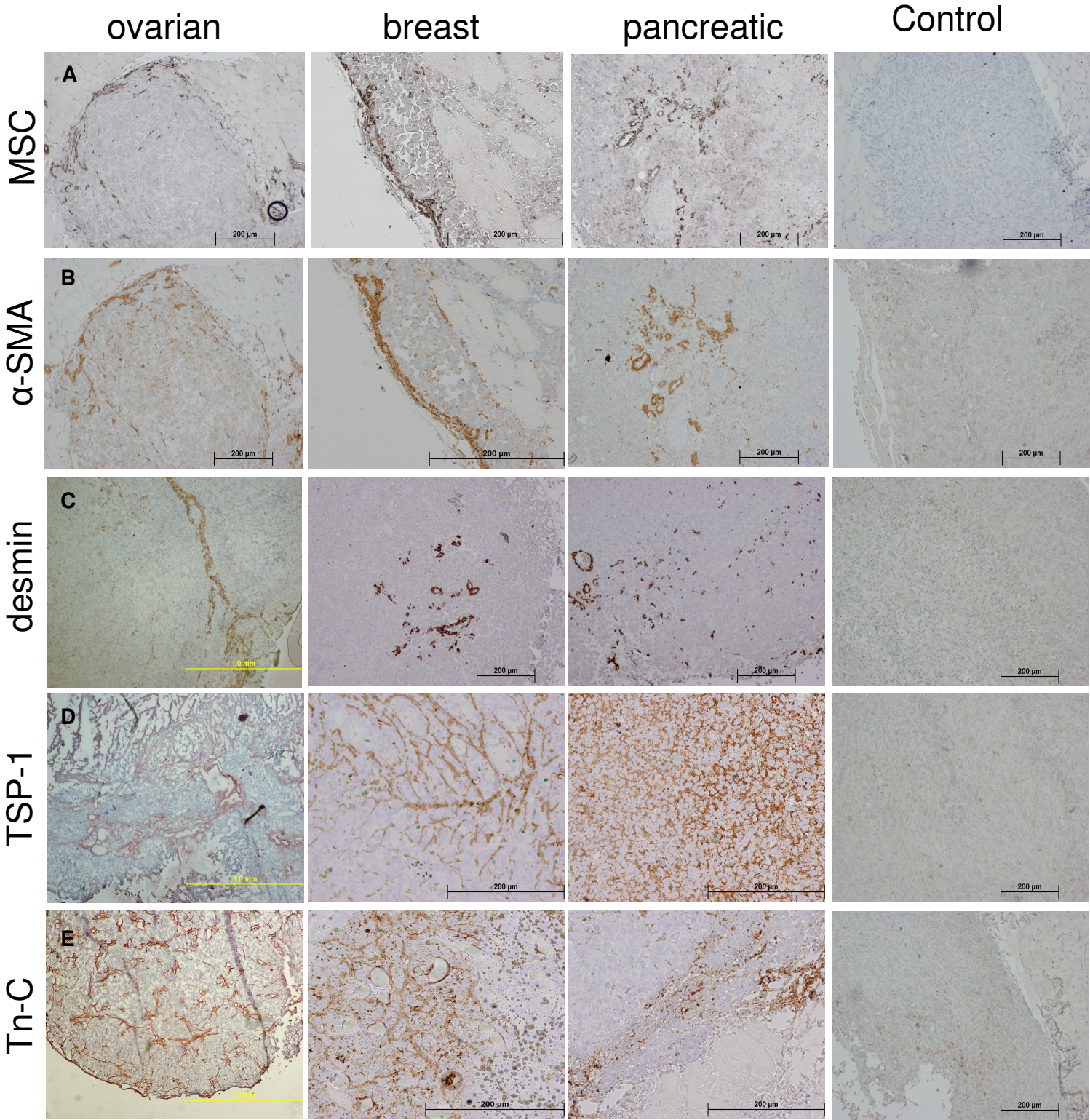

Supplement: Figure S3 — In ovarian, breast, and pancreatic xenograft mouse models, tumors show patterns of fibrovascular networks following intravenous injection of huMSC. Tumor cells, were allowed to engraft prior to administration of huMSC [4 times, once per week, Skov-3 (IP), MDA-231 (IV) or Panc-1 (IV)]. On week 14, mice were sacrificed and tumors for histology were collected. (A) We stained for the presence of MSC (Thy-1) which we showed co-localized expression with α-SMA on adjacent tissue sections. (B) Further tissue sections are not adjacent sections. All three tumor types show fibrovascular network pattern staining of α-SMA, (C) desmin, (D) Tn-C, and (E) TSP-1 within the tumors that were treated with MSC while tumors not treated with MSC showed no staining (negative tumor, Skov-3-only, is representative of MDA and Panc-1 tumors). (6.51 MB PDF) [file pone.0004992.s003.pdf]

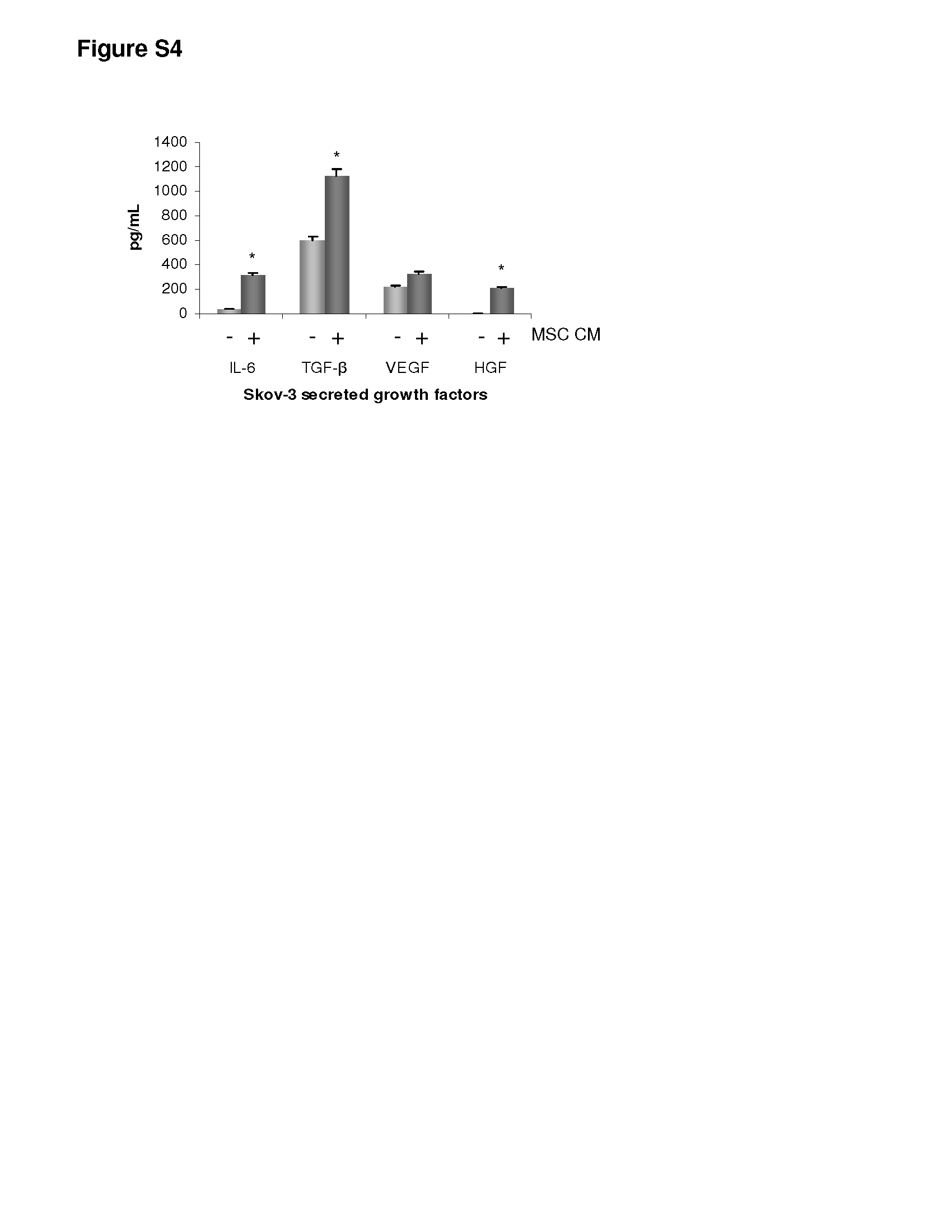

Supplement: Figure S4 — Skov-3 tumor cell secretion of growth factors following the stimulation with MSC-CM. Secreted proteins are measured from Skov-3 tumor cell cultures prior to and post stimulation with MSC-CM. IL-6 (P<0.0001), VEGF (P<0.01), HGF (P<0.0001) and TGF-β (P<0.001) secretion are all increased following conditioning with MSC CM. (0.34 MB TIF) [file pone.0004992.s004.tif]
